# Are *Ureaplasma* spp. a Cause of Nongonococcal Urethritis? A Systematic Review and Meta-Analysis

**DOI:** 10.1371/journal.pone.0113771

**Published:** 2014-12-02

**Authors:** Nan Zhang, Rong Wang, Xue Li, Xu Liu, Zhaobing Tang, Yunde Liu

**Affiliations:** 1 Department of Clinical Laboratory, the First Teaching Hospital of Tianjin University of Traditional Chinese Medicine, Tianjin, China; 2 Division of Clinical Microbiology, School of Laboratory Medicine, Tianjin Medical University, Tianjin, China; 3 Department of Urology, the First Affiliated Hospital, Chongqing Medical University, Chongqing, China; University of Utah School of Medicine, United States of America

## Abstract

**Background:**

Nongonococcal urethritis (NGU) is the most common male reproductive tract syndrome. *Ureaplasmas* spp. including *U. urealyticum* and *U. parvum*, have been increasingly reported to be implicated in NGU. However, there are still many contradictions about their pathogenic role in NGU.

**Aims:**

The goals of this study were to evaluate the association of *Ureaplasmas* spp. with NGU, and to compare the prevalence of *Ureaplasmas* spp. infection in China relative to the world average.

**Methods:**

A systematic review and meta-analysis was conducted following standard guidelines for meta-analysis. The quality of included studies was assessed by Newcastle-Ottawa scale.

**Results:**

A total of seven studies involving 1,507 NGU patients and 1,223 controls were eligible for meta-analysis. There was no significant difference in the *Ureaplasma* spp. positive rate between the NGU and control groups. However, the *U. urealyticum* positive rate was significantly higher in NGU patients compared to controls; the *U. parvum* positive rate was significantly higher in controls compared to NGU patients. Furthermore, within the NGU patient group, the positive rate of *U. urealyticum* was significantly higher than that of *U. parvum*, whereas within the control group, the opposite trend was observed. Compared to the world average, a significantly higher positive rate of *Ureaplasma* spp. was observed in both the NGU and control groups in China.

**Conclusions:**

Our analysis supports that *U. urealyticum*, but not *U. parvum*, is an etiological agent in NGU. More detailed studies of these two species in China and the world could contribute to a better understanding of the epidemiology and pathogenesis, and facilitate the development of better strategies for treatment and prevention of NGU.

## Introduction

Nongonococcal urethritis (NGU), a clinical syndrome consisting of urethral manifestations such as urethral discharge, dysuria and irritation, in the absence of gonococcal infection, is the most common urethritis syndrome observed in men. In general, NGU accounts for 20–50% of urethritis cases among men in sexually transmitted disease (STD) clinics [1], [2]. While there are a number of known pathogens contributing to NGU, including *Chlamydia trachomatis* (20–50%), *Mycoplasma genitalium* (10–25%), and *Trichomonas vaginalis* (5–15%) [2], [3], as many as 45% of NGU cases are of unknown etiology [2], [4]. There have been reports of several other microbes associated with NGU, but their role has not been proven. Among such microbes, *Ureaplasmas* spp. are perhaps the most extensively studied and the most controversial organisms.

Since *Ureaplasmas* spp. were first isolated in 1954 from the urethral discharge of men with NGU [5], many investigators have attempted to determine whether these organisms are a cause of the disease. Early studies from the 1960 s to 1990 s [6]–[9] showed conflicting results on the association of *Ureaplasmas* spp. with NGU, which led to the speculation of existence of different ureaplasma strains or biovars with distinct virulence potential. In 1999, phylogenetic analysis established the classification of *Ureaplasma* spp. into two species, *U. parvum* and *U. urealyticum* [10], which raised the question of whether the conflicting results of early studies were due to the lack of differentiation between *U. parvum* and *U. urealyticum.* Since then, intense efforts have been made in differential detection of these two species in NGU patients [11]–[13]. Several studies based on differential detection have suggested that *U. parvum* is a non-pathogenic commensal organism in the male urethra [3], [14], [15], which presumably account for the conflicting results of early studies. Nonetheless, the epidemiologic data regarding the association of differentiated *U. parvum* and *U. urealyticum* with NGU remain unclear [3], [4], [13]–[15]. The disagreement among different studies may be attributable in part to the choice of patient population and inclusion criteria, and the restricted sample size of the patients in individual studies, which may limit the power of the statistical tests.

China is one of the most populated countries in the world. Over the past two decades, rapid pace of economic and social changes has been followed by a resurgent epidemic of STDs [16], [17]. Since 1991, NGU has surpassed gonorrhoea and becomes among the top of the eight common reportable STDs in China [18]. Based on a cross-sectional study of STD clinic attendees in two disparate Chinese cities, the most common diagnosis in STDs was NGU (22.2%), followed by genital warts (13.2%), syphilis (11.6%), and gonorrhoea (8.4%) [19]. The known pathogens associated with NGU in China mainly include *C. trachomatis* (15.5–41.4%) [20], [21], *M. genitalium* (6.9–25%) [22], [23], and *T. vaginalis* (9.7%) [24]. The incidence of undifferentiated *Ureaplasma* spp. infection in NGU patients varies from 31.6% to 43.8% [21], [25]. There are few reports available regarding the prevalence of differentiated *U. parvum* and *U. urealyticum* and their association with NGU [26]–[28].

To better understand the role of *U. parvum* and *U. urealyticum* in NGU, we performed a comprehensive meta-analysis of literature published between January 2000 and December 2013 about the association of *U. parvum* and *U. urealyticum* with NGU in patients in China and worldwide.

## Methods

### Literature search

A meta-analysis review was conducted according to the PRISMA Statement [29]. We used the following biomedical literature databases: PubMed, Embase, Wangfang (Chinese), VIP (Chinese) and Chinese National Knowledge Infrastructure (CNKI). The keywords used for literature search were “Nongonococcal urethritis OR nongonococcal urethritis OR NGU AND *Ureaplasma* spp. OR *U. urealyticum* OR *U. parvum*”. Given that *U. parvum* was not recognized as a new species until 1999, the literature search period was set from January 2000 to December 2013 (prior to the preparation of this manuscript). There was no language restriction in our selection. Additional publications were identified by manual search of the references of the publications obtained by computer search. When there were publications with duplicated or overlapped data from the same investigators, only one of the most recent or complete studies were used.

### Inclusion criteria

The inclusion criteria for NGU were adapted from published studies [4], [30], including visible urethral discharge on physical examination and/or microscopic evidence of urethritis with ≥5 polymorphonuclear leukocytes (PMNs) per high-powered field (HPF; 10003) averaged ≥3 fields on a urethral gram-stained smear. All control subjects had no noticeable urethral symptoms or signs. The eligible studies that were included in the meta-analysis met the following criteria: (a) the studies were case-control studies designed to explore the association of *Ureaplasma* spp., *U. urealyticum* or *U. parvum* with NGU; (b) the patient group was men who were diagnosed with NGU; (c) the studies provided enough data for the estimation of odds ratios (ORs) with 95% confidence intervals (CI).

### Data extraction

Three reviewers (Nan Zhang, Rong Wang and Xue Li) independently searched the databases described above. We first selected articles on the basis of the title and abstract. Data were extracted using a standardized extraction form. If any of the three reviewers considered any article to be potentially eligible, the full-text of the article was retrieved and evaluated by all three reviewers using the above inclusion criteria. Discordance among reviewers regarding articles with borderline eligibility was resolved by consensus following discussion with an additional investigator (Yunde Liu). For each of the selected articles, the following information was recorded: name of the first author, year of publication, country of origin of study population, total number of patient cases and controls, and number of cases infected with *U. urealyticum* or *U. parvum*.

### Assessment of study quality

The quality of the included studies was assessed using the Newcastle-Ottawa scale [31]. The scale consists of nine items that cover three dimensions: a) selection, total score: 4; b) comparability, total score: 2; and c) exposure (case-control)/outcome (cohort), total score: 3. A high score out of a total of nine points indicates high quality.

### Statistical analysis

The association of *Ureaplasma* spp., *U. urealyticum* or *U. parvum* with NGU was calculated using ORs and 95% CIs. The distribution rates of *U. urealyticum* and *U. parvum* within the control and NGU groups were also compared using ORs and 95% CIs. The statistical significance of the summary OR was determined using the Z test, with *P*<0.05 considered statistically significant. Statistical heterogeneity between studies was evaluated using the *χ^2^* and *I^2^* tests with significance level set at *P*<0.1 or *I^2^*>50% [32]. If heterogeneity existed, the data were analysed using a random effects model. In the absence of heterogeneity, a fixed effects model was used. The Begg’s rank correlation was used to assess the potential publication bias. The difference in prevalence between China and the world was assessed by *chi-square* test, with *P*<0.05 considered statistically significant. All statistical analyses were conducted using Stata software (version 11.0; Stata Corporation, College Station, TX) and two-sided *P* values.

## Results

### Study selection and study characteristics

**Figure 1:**
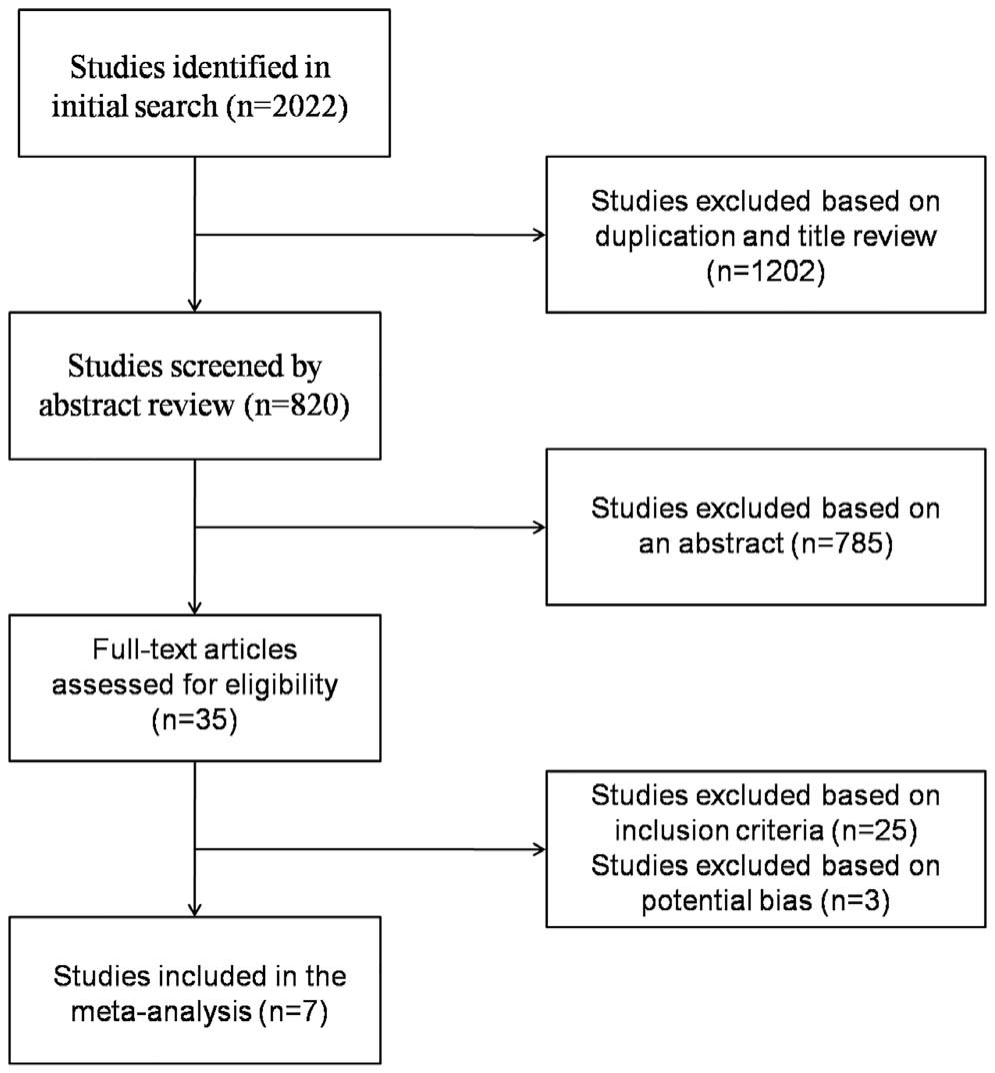
Flow diagram of the literature search process.

There were a total of 2,022 relevant articles retrieved (Fig. 1). After removal of duplicates and screening of titles, 820 articles were left and further reviewed based on the abstract. There were 785 articles excluded based on abstract review. The remaining 35 articles were evaluated based on the full text; 10 of them were selected based on the inclusion criteria, which are listed in Table 1. Three of the 10 articles were excluded because in these articles the control group did not match the NGU group in the population characteristics [27], [28] or the method to detect *Ureaplasma* spp. was culture-based and unable to differentiate *U. urealyticum* and *U. parvum* [28], [33]. Finally, there were 7 studies [3], [4], [13], [14], [26], [34], [35] that met all search criteria and were included for meta-analysis.

**Table 1:** Summary of case-controlled studies included in this report.

Study no.	Author	Publicationyear	Country	Design	Age (year)	NGU Group		Control Group		Quality assessment ofstudies according toNewcastle-Ottawa Scale			
						No. of *Ureaplasma*spp. positive cases(co-infection cases)	Samplesize (n)	No. of *Ureaplasma*spp. positive cases(co-infection cases)	Sample size(n)	Selection	Comparability	Exposure	Total
1*	Povlsen et al. [14]	2002	Sweden	Case-control	NGU: 25^ a^;Control: 27^ a^	39(1)	125	60(2)	205	3	0	3	6
2*	Yoshida et al. [34]	2005	Japan	Case-control	16–65 yrs (24^ b^)	77	317	30	141	4	0	2	6
3*	Zhou et al. [26]	2005	China	Case-control	18–61 yrs (35^ b^)	19	36	14	33	3	1	3	7
4*	Bradshaw et al. [3]	2006	Australia	Case-control	32.3±9.1^ b^	66	329	98	307	4	1	2	7
5*	Couldwell et al. [35]	2010	Australia	Case-control	NGU: 35.9^ b^Control: 31.5^ b^	48	268	35	237	4	1	3	8
6*	Ondondo et al. [13]	2010	United States	Case-control	16–49	48	119	55	117	4	1	3	8
7*	Wetmore et al. [4]	2011	United States	Case-control	≥16	128(4)	313	86(5)	183	4	1	3	8
8	Horner et al. [33]	2001	England	Case-control		33	109	10	62				
9	Jiang et al. [27]	2006	China	Case-control		40	100	46	98				
10	Wang et al. [28]	2007	China	Case-control		16	28	43	136				

*Included for meta-analysis. ^a^Median age. ^b^Mean age.

The seven included studies involved a total of 1,507 NGU patients and 1,223 controls (Table 1). The number of *Ureaplasma* spp. positive cases was 425 in the NGU group (271 cases of *U. urealyticum*, 149 cases of *U. parvum* and 5 cases infected with both species), and 378 in the control group (161 cases of *U. urealyticum*, 210 cases of *U. parvum* and 7 cases infected with both). All study subjects involved were hospital-based. Data about demographic and sexual behavioural characteristics were available from only some but not all of the studies, and thus were not included for meta-analysis.

### Quantitative synthesis

#### Comparison I: Association of undifferentiated *Ureaplasma* spp. with NGU

**Figure 2:**
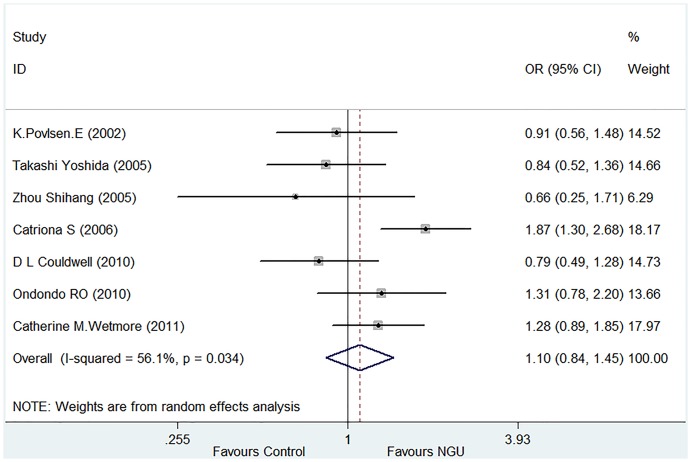
Forest plot for the meta-analysis of the association of undifferentiated *Ureaplasma* spp. with NGU.

By including a total of 1,507 patients with NGU and 1,223 controls from seven eligible studies (Table 1), meta-analysis demonstrated no significant difference in the positive rate of undifferentiated *Ureaplasma* spp. infection between the NGU patients (28.20%) and control groups (30.91%) (Fig. 2). The pooled *OR* was 1.10 (95% *CI*: 0.84–1.45) with *Z* = 0.69 and *P* = 0.488 for the overall effect. In the statistical heterogeneity analysis, *P* value was 0.034 and *I^2^* value was 56.1%. These data suggest that undifferentiated *Ureaplasma* spp. are not associated with NGU.

#### Comparison II: Association of differentiated *U. urealyticum* and *U. parvum* with NGU

**Figure 3:**
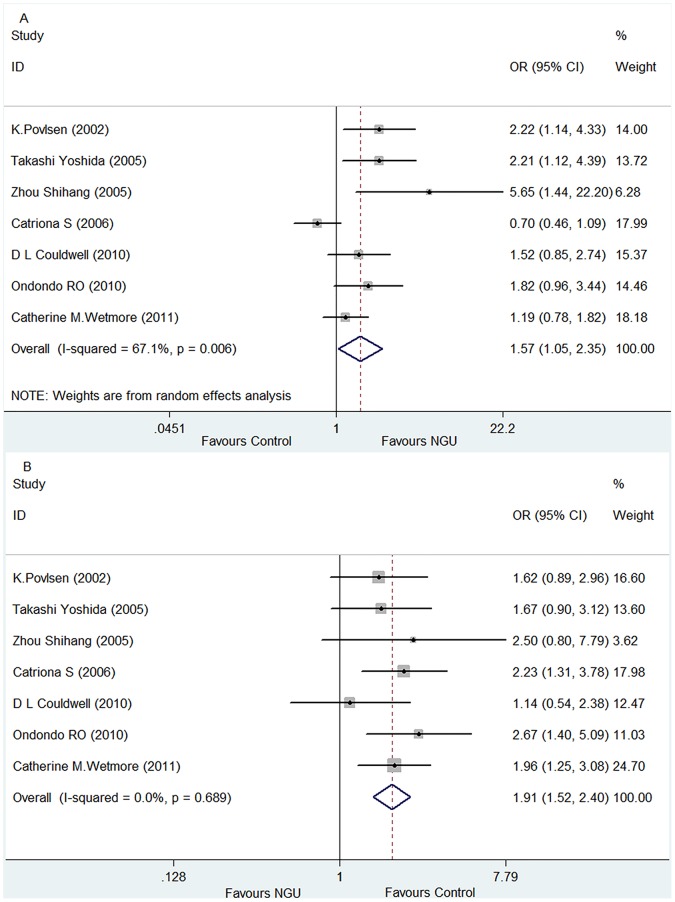
Forest plots for the meta-analysis of the association of differentiated *U. urealyticum* and *U. parvum* with NGU. (A) Comparison of the *U. urealyticum* infection rate between the NGU and control groups. (B) Comparison of the *U. parvum* infection rate between the NGU and control groups.

By comparing the *U. urealyticum* positive rate between the NGU patients (276/1,507 or 18.31%) and the controls (168/1,223 or 13.74%), meta-analysis demonstrated a significant difference between these two groups with a pooled *OR* of 1.57 (95% *CI*: 1.05–2.35), and *Z* = 2.19 and *P* = 0.029 for the overall effect (Fig. 3A). In the heterogeneity test, *P* value was 0.006 and *I^2^* value was 67.1%. The finding of a significantly higher prevalence of *U. urealyticum* in NGU patients than in the controls supports a positive association of this organism with NGU.

By comparing the *U. parvum* positive rate between the NGU patients (154/1,507 or 10.22%) and control groups (217/1,223 or 17.74%), meta-analysis demonstrated a significant difference between these two groups, with a pooled *OR* value of 1.91 (95% *CI*: 1.52–2.40), and *Z* = 5.52 and *P*<0.00001 for overall effect. In the heterogeneity test, *P* value was 0.689 and *I^2^* value was 0%. The forest plot is shown in Fig. 3B. The finding of a significantly lower prevalence of *U. parvum* in NGU patients than in the controls rejects an association of this organism with NGU.

#### Comparison III: Distribution of *U. urealyticum* and *U. parvum* within the NGU and control groups

**Figure 4:**
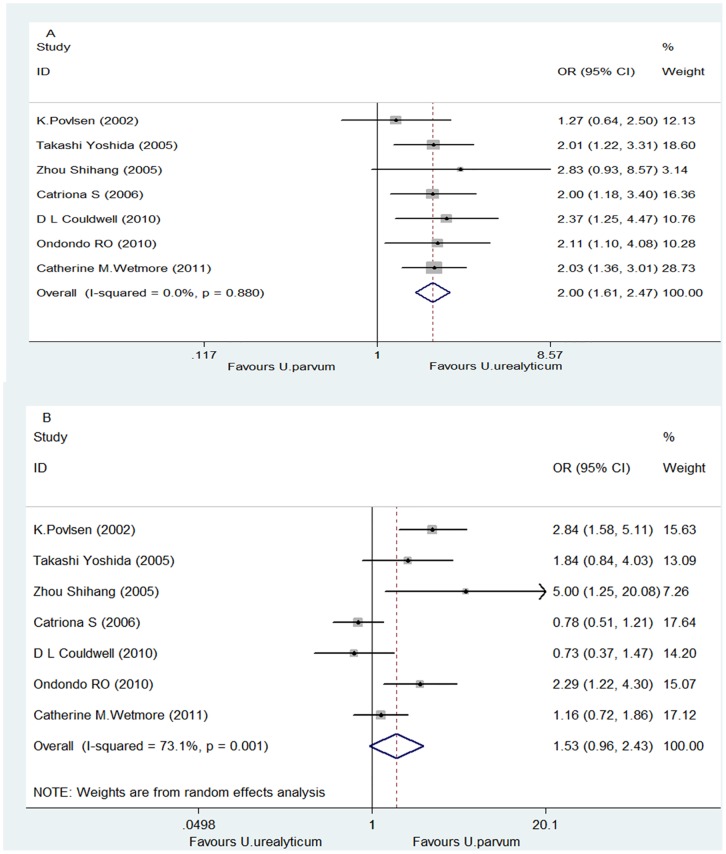
Forest plots for the meta-analysis of the distribution of *U. urealyticum* and *U. parvum* within the NGU and control groups. (A) The distribution of *U. urealyticum* and *U. parvum* within the NGU group. (B) The distribution of *U. urealyticum* and *U. parvum* within the control group.

By comparing the distribution of *U. urealyticum* and *U. parvum* within the 1,507 NGU patient group, the prevalence of *U. urealyticum* (18.31%) was significantly higher than that of *U. parvum* (10.22%), with a pooled *OR* value of 2.00 (95% *CI*: 1.61–2.47), *Z* = 6.34 and *P*<0.00001 for the overall effect, and *P* = 0.880 and *I^2^* = 0% for the heterogeneity test (Fig. 4A).

By comparing the distribution of *U. urealyticum* and *U. parvum* within the 1,223 controls, the prevalence of *U. urealyticum* (13.74%) was significantly lower than that of *U. parvum* (17.74%), with a pooled *OR* value of 1.53 (95% *CI*: 0.96–2.43), Z = 1.79 and *P* = 0.074 for the overall effect, and *P* = 0.001 and *I^2^* = 73.1% for the heterogeneity test (Fig. 4B).

#### Comparison IV: Prevalence of undifferentiated *Ureaplasma* spp. in NGU in the world and China

Of the seven studies used for meta-analysis, only one was from China [26], in which the prevalence of *U. urealyticum* was significantly higher in NGU patients than controls (36% vs. 9%, *P* = 0.01) while the prevalence of *U. parvum* was not (17% vs. 33%, *P* = 0.162). The small sample size from this single study precludes meaningful comparison of the prevalence of these two species between China and the world. There were two other case-controlled studies [27], [28] from China excluded for meta-analysis because in both studies the participants did not met all the inclusion criteria while in one study *U. urealyticum* was not differentiated from *U. parvum* [28]. However, both studies reported the prevalence of *Ureaplasma* spp. in a total of 128 NGU patients and 234 controls. Therefore, we combined these three studies[26]–[28] from China to estimate the prevalence of undifferentiated *Ureaplasma* spp. in China and compared to the global data. One case-control study from England [33], which was excluded for meta-analysis due to failure to meet all the inclusion criteria, was included in this comparative analysis in order to increase the sample size and geographic representation for the global data. As shown in Table 2, there was no significant difference in the prevalence of *Ureaplasma* spp. between the NGU and control groups in China (45.73% vs. 38.58%, *χ^2^* = 2.145, *P* = 0.143. Table 2). However, the prevalence of *Ureaplasma* spp. in both the NGU and control groups was significantly higher in China than in the world (*χ^2^* = 18.57 and *P*<0.0001 for the NGU group; *χ^2^* = 5.330 and *P* = 0.021 for the control group. Table 2).

**Table 2:** The prevalence of undifferentiated *Ureaplasma* spp. in NGU in China and the world.

	NGU group		Control group	
	China	World	China	World
Positive case	75	514	103	477
Sample size(n)	164	1744	267	1519
positive rate^a^	45.73%	29.47%	38.58%	31.40%

a *χ^2^* = 1.430, *P* = 0.232 between the NGU and control groups in the world.

*χ^2^* = 2.145, *P* = 0.143 between the NGU and control groups in China.

*χ^2^* = 18.57, *P*<0.0001 for the NGU group between China and the world.

*χ^2^* = 5.33, *P* = 0.021 for the control group between China and the world.

### Publication bias

**Figure 5:**
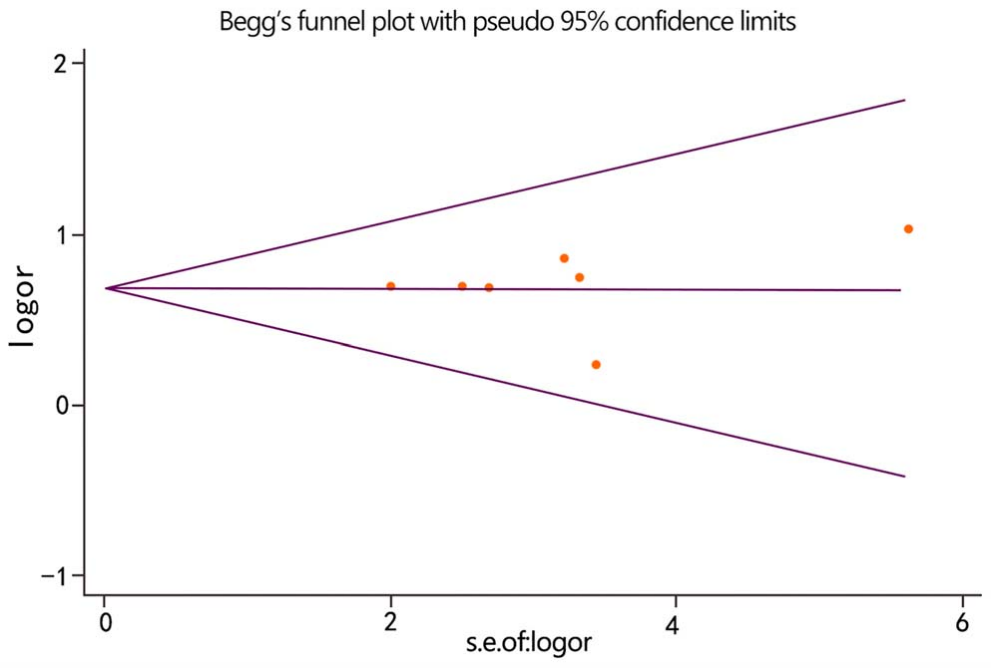
Funnel plot for the meta-analysis of the distribution of *U. urealyticum* and *U. parvum* within the NGU group. The horizontal line represents the natural log (ln) of the combined OR. The funnel lines represent the pseudo 95% confidence limit.

For each meta-analysis, publication bias was assessed by the funnel plot and Begg’s test. Fig. 5 shows the results of funnel plot for the meta-analysis of the distribution of *U. urealyticum* and *U. parvum* within the NGU group. The plot appeared to be approximately symmetrical and the Begg’s test was not statistically significant (*P* = 0.764), suggesting no publication bias existed. However, we found potential publication bias in the comparison of the *U. urealyticum* infection rate between the NGU and control group (Begg’s test *P* = 0.016). No potential publication bias was detected in the other meta-analysis. The Begg’s test showed *P* values of 0.540, 0,881 and 0.368, respectively, for comparison of the *Ureaplasma* spp. prevalence between the NGU and control groups, the *U. parvum* prevalence between the NGU and control groups, and the prevalence of *U. urealyticum* and *U. parvum* within the control group.

## Discussion

There has been a great deal of disagreement about the role of *Ureaplasma* spp. in NGU. While there are numerous epidemiologic data collected from various study populations examined for either undifferentiated *Ureaplasma* spp. or differentiated *U. parvum* and *U. urealyticum*, it remains inconclusive whether undifferentiated or differentiated species play a pathogenic role in NGU. To summarize the results of and to overcome small sample sizes of existing individual studies, we performed this meta-analysis. To the best of our knowledge, this is the first meta-analysis to assess the association of *U. urealyticum* and *U. parvum* with NGU.

Our meta-analysis involves a total of 1,507 patients with NGU and 1,223 controls from seven eligible studies [3], [4], [13], [14], [26], [34], [35] originated from 5 different countries or 4 different continents (Asia, North America, Europe and Australia. Table 1). This meta-analysis resulted in the following findings. Firstly, there was no significant difference in the prevalence of undifferentiated *Ureaplasma* spp. infection between the NGU and control groups (28.20% vs. 30.91%. Fig. 2), suggesting that undifferentiated *Ureaplasma* spp. are not associated with NGU. Secondly, the prevalence of *U. urealyticum* infection was significantly higher in NGU patients than in controls (18.31% vs.13.74%. Fig. 3A), suggesting an association of this organism with NGU. This finding is further supported by the significantly higher prevalence of *U. urealyticum* infection than that of *U. parvum* infection within the NGU group (18.31% vs. 10.22%. Fig. 4A) as well as the significantly lower prevalence of *U. urealyticum* infection than that of *U. parvum* infection within the control group (13.74% vs. 17.74%. Fig. 4B). Thirdly, the prevalence of *U. parvum* infection was significantly lower in NGU patients than in the controls (10.22% vs. 17.74%. Fig. 3B), suggesting no association of this organism with NGU. This finding is further supported by the results of the comparison of the distribution of *U. urealyticum* and *U. parvum* infections within the control group as described above. Altogether, our meta-analysis suggests that the association of *ureaplasmas.*spp with NGU depends on the species detected and that *U. urealyticum*, but not *U. parvum*, is an etiological agent in NGU.

Our findings of the association of *U. urealyticum* with NGU are consistent with most of the previous epidemiologic studies of differentiated *ureaplasma* species in NGU patients, including all the 7 studies selected for the meta-analysis except for only one [3] in which neither *U. urealyticum* or *U. parvum* was found to be associated with NGU. The association of *U. urealyticum* with NGU is further supported by the following observations in previous studies: I) Significant association of *U. urealyticum* organism loads with the development of NGU [36]; II) The presence of elevated antibody responses in patients infected with *U. urealyticum* [6], [37]; III) Experimental infection of BALB/c mice by intraurethral inoculation of *U. urealyticum* organisms [21]; IV) Successful reproduction of symptomatic urethritis in human volunteers following experimental intraurethral inoculation of *U. urealyticum* organisms [38]. Based on all these data, it seems rational to conclude that *U. urealyticum* has a pathogenic role in NGU.

Our findings of no association of *U. parvum* with NGU are consistent with most of the previous epidemiologic studies of differentiated ureaplasmas species in NGU patients, including all the 7 studies selected for the meta-analysis [3], [4], [13], [14], [26], [34], [35], in which the prevalence of *U. parvum* in NGU patients was very similar or even significantly lower compared to controls. These data support the notion that *U. parvum* is a non-pathogenic commensal organism in the male urethra [3], [14], [15]. The detection of the high prevalence of *U. parvum* in controls may explain why undifferentiated *Ureaplasma* spp. are not associated with NGU in some of the early studies [2]. However, given that considerable studies have shown potential associations of *U. parvum* with various diseases including adverse pregnancy outcomes [39], cervicitis [20] and prosthetic joint infection [22], the possibility cannot be ruled out that only certain subtypes of *U. parvum* are association with NGU or other diseases as has been suggested from a recent study by De Francesco et al. [40], who found that *U. parvum* 3/14 and T960 biovar were significantly associated with genital infections.

Despite a continuing increase in the prevalence of STDs in China [17], [18], there have been very few case-controlled studies of the prevalence of *Ureaplasma* spp. and their association with NGU. In this report, we searched the literature and found only three case-controlled studies [26]–[28], involving a total of 164 NGU patients and 267 controls. Our analysis showed no significant difference in the prevalence of *Ureaplasma* spp. between the NGU and control groups (Table 2), suggesting no association of this organism with NGU, consistent with the global data described above. Strikingly, the prevalence of *Ureaplasma* spp. in both the NGU and control groups in China was significantly higher in comparison with the global data set, especially in the NGU group (45.73% vs. 29.47%, *P*<0.0001. Table 2). This may be an important observation and needs to be explored further by investigating differentiated *U. urealyticum* and *U. parvum* species in large and geographically diverse patient populations in China. It would be of interest to look at the strain types of *U. urealyticum* and *U. parvum* in China and the possibility of a higher susceptibility of Chinese people to these species.

Meta-analysis is a powerful statistical method and has the capability to increase the sample size and thus potentially to improve the power to assess the association of a pathogen with a disease both qualitatively and quantitatively. However, there are some limitations to its use in the present study. Firstly, the main limitation of this meta-analysis is that only a small number of studies were retrieved, with more than a half of the total participants originated from the United States and Australia, and thus they may not represent sufficient diversity of the patient populations. Additionally, the possibility exists that some studies with negative association results are not published and thus not retrievable, which may lead to publication biases in the meta-analysis. Secondly, the inherent heterogeneity of the included studies, in terms of study population and design, detection method, and data interpretation, rendered it difficult to integrate results across studies. Particularly, some of the included studies did not provide details about the patient sociodemographic or sex behavioral characteristics, or if patients were co-infected with other known pathogens associated with NGU, especially *C. trachomatis*, *M. genitalium* and *T. vaginalis*. The lack of analysis of these confounding factors may weaken the measures of association of *U. urealyticum* and *U. parvum* with NGU. Thirdly, all the seven studies included in our meta-analysis are case-controlled without longitudinal data, thus precluding any conclusions about trends of the prevalence of *U. urealyticum* and *U. parvum* in NGU.

In conclusion, our meta-analysis supports that *U. urealyticum*, but not *U. parvum*, is an etiological agent in NGU. More detailed studies of these two species in China and the world could contribute to a better understanding of the epidemiology and pathogenesis, and facilitate the development of better strategies for treatment and prevention of NGU.

## Supporting Information

Checklist S1 **PRISMA checklist.**
(DOC)
